# An endobronchial ultrasound scope enables endoscopic ultrasound-guided tissue acquisition for pancreatic lesions in a patient with trismus

**DOI:** 10.1055/a-2944-3742

**Published:** 2026-09-11

**Authors:** Daiki Agarie, Hirofumi Miyahira, Reo Tome, Shigehito Tameda, Shingo Arakaki, Tatsuji Maeshiro, Kazuko Yamamoto

**Affiliations:** 1 First Department of Internal Medicine, Division of Infectious, Respiratory, and Digestive Medicine 118113 University of the Ryukyus HospitalGinowanOkinawaJapan; 2 Department of Diagnostic Pathology 118113 University of the Ryukyus HospitalGinowanOkinawaJapan

Endoscopic ultrasound-guided tissue acquisition (EUS-TA) is a well-established technique for diagnosing pancreatic cancer, offering high diagnostic accuracy and safety. 1​ However, conventional gastrointestinal echoendoscopes have a larger outer diameter than standard upper gastrointestinal endoscopes, making insertion difficult in patients with trismus or esophageal strictures. We report a case in which tissue acquisition was successfully achieved using an endobronchial ultrasound (EBUS) scope, despite the inability to insert a conventional echoendoscope ( Video 1 ).

**Video 1** EUS-TA using an endobronchial ultrasound scope for pancreatic body cancer in a patient with trismus.


**Fig. 1:**
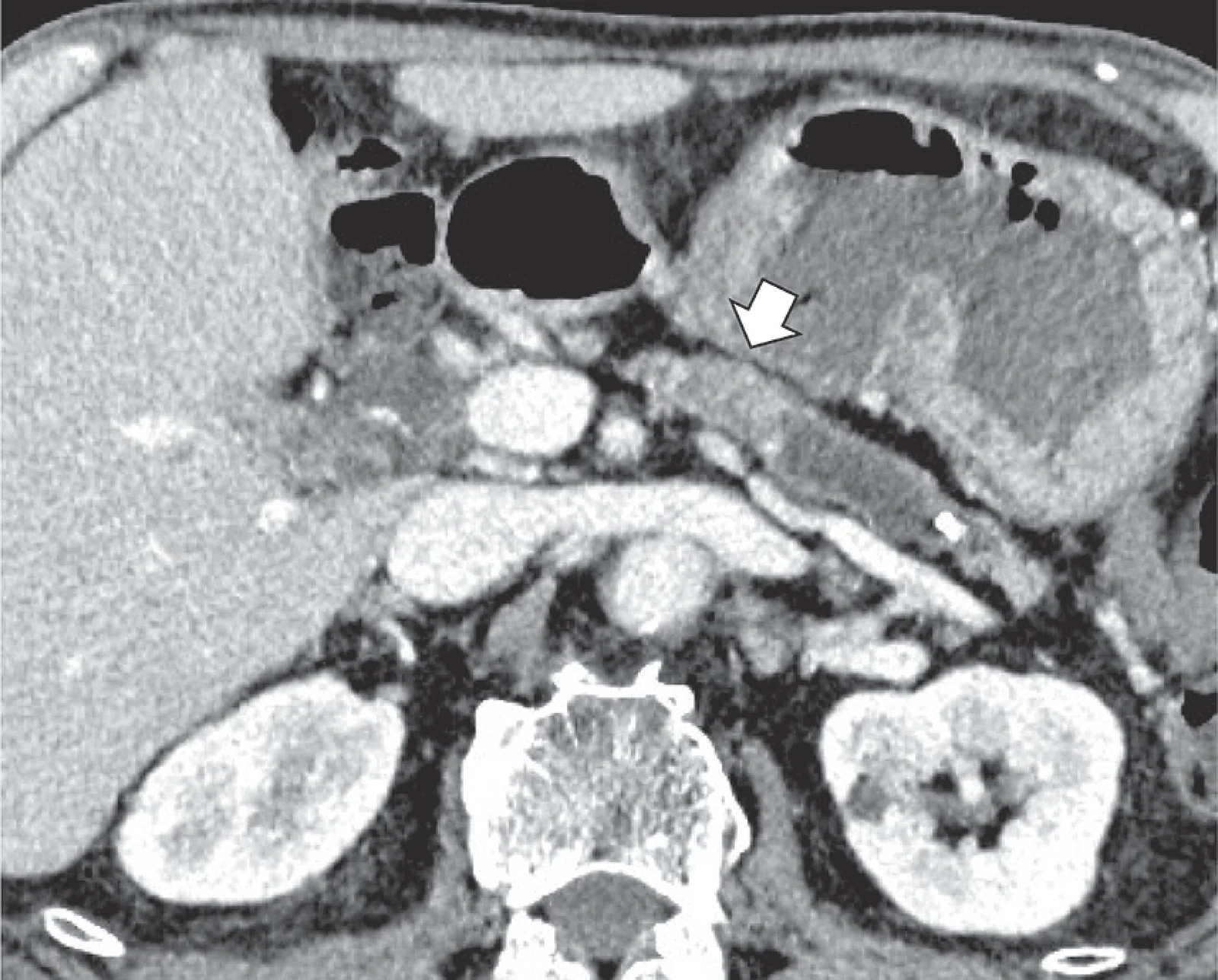
Contrast-enhanced computed tomography revealed a hypovascular mass in the pancreatic body (white arrow) with dilation of the distal pancreatic duct.

**Fig. 2:**
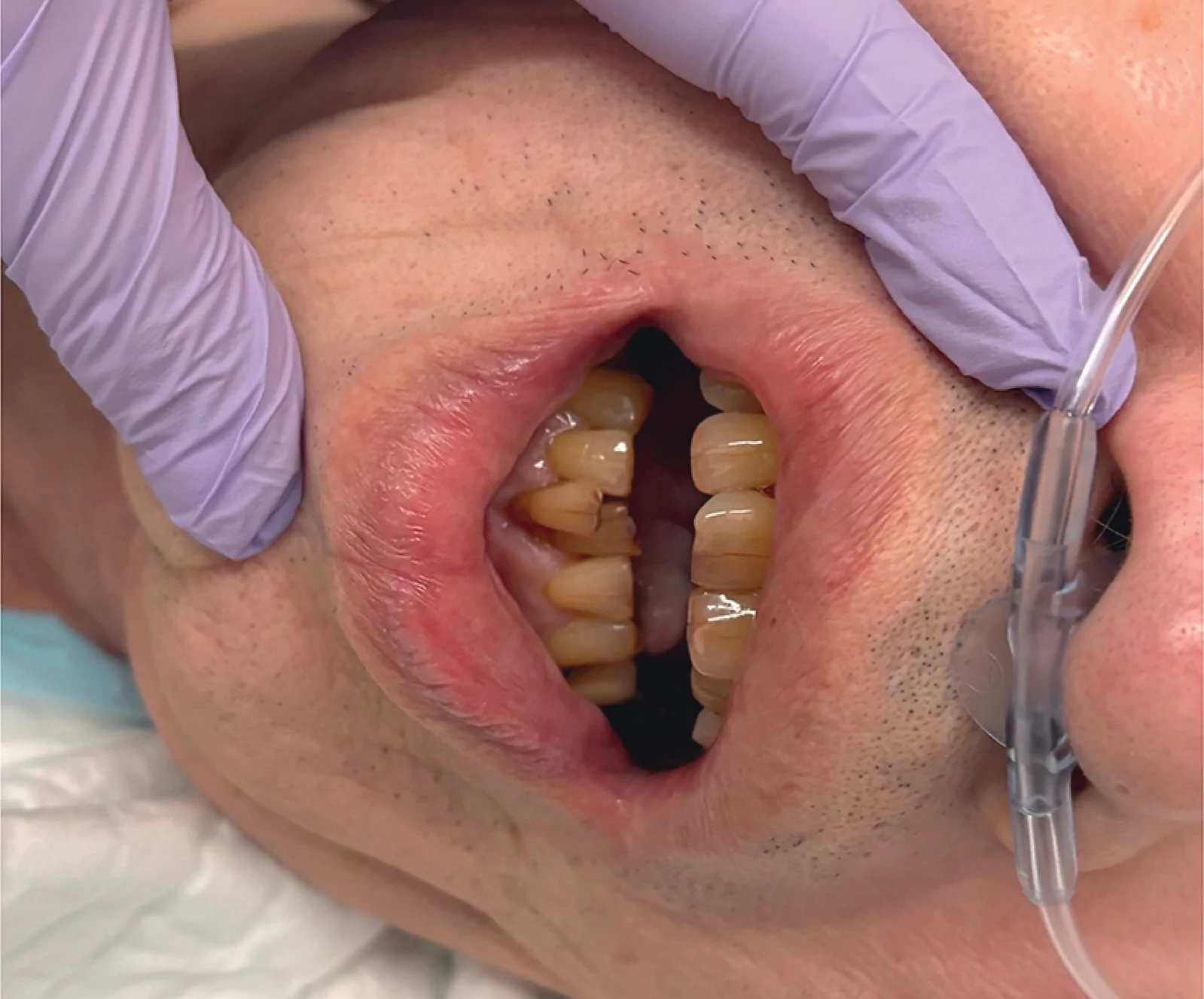
Postoperative trismus following tongue cancer surgery severely limited mouth opening to approximately 10 mm.

**Fig. 3:**
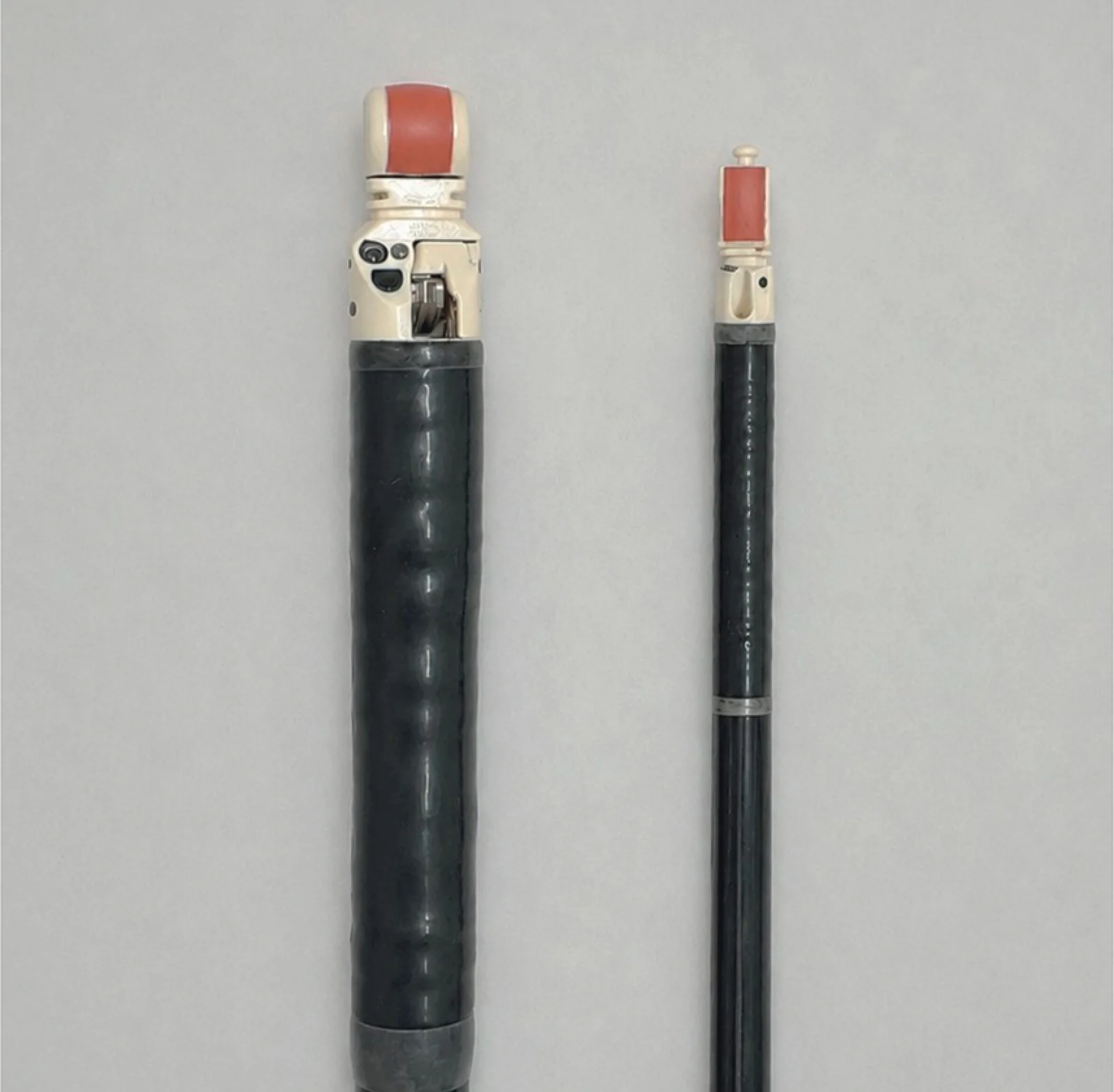
The conventional echoendoscope (GF-UCT260, Olympus, Tokyo, Japan; left) has a distal end outer diameter of 14.6 mm. The endobronchial ultrasound (EBUS) scope used in this case (BF-UC260FW, Olympus, Tokyo, Japan; right) has a distal end outer diameter of 6.9 mm, enabling transoral insertion in this patient with severe trismus.

A 75-year-old man with a history of partial glossectomy and cervical lymph node dissection for tongue cancer was referred for the evaluation of a pancreatic body mass detected on contrast-enhanced computed tomography ( Fig. 1 ). EUS-TA was planned; however, due to postoperative trismus, mouth opening was restricted to approximately 10 mm ( Fig. 2 ), preventing the insertion of a conventional echoendoscope (GF-UCT260, Olympus, Tokyo, Japan). Therefore, EUS-TA was performed using an EBUS scope (BF-UC260FW, Olympus, Tokyo, Japan; Fig. 3 ).

**Fig. 4:**
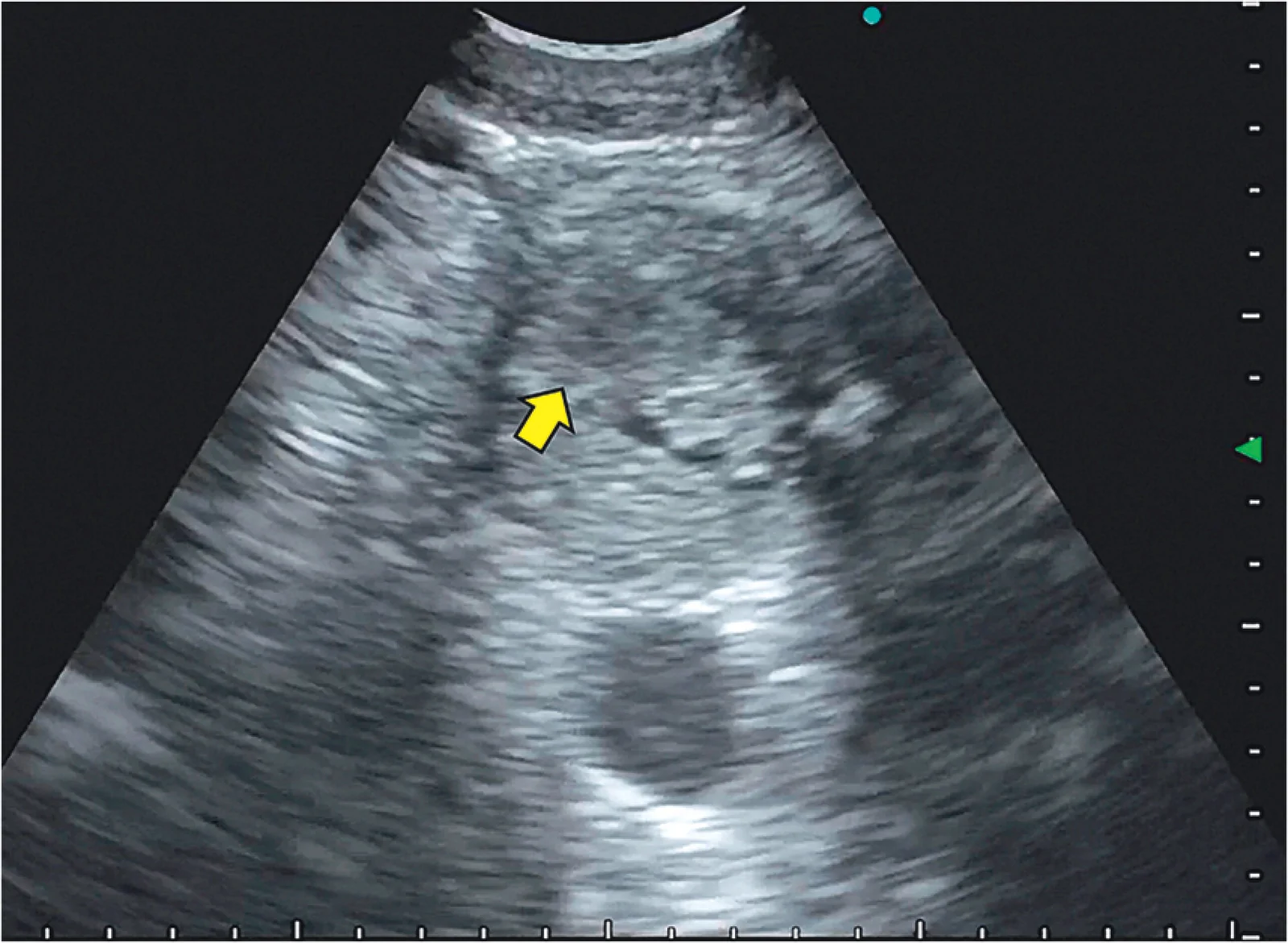
An endoscopic ultrasound image of the lesion. A papillary-growing tumor was identified within the main pancreatic duct. A focal hypoechoic change in the surrounding parenchyma raised suspicion for invasion (yellow arrow).

**Fig. 5:**
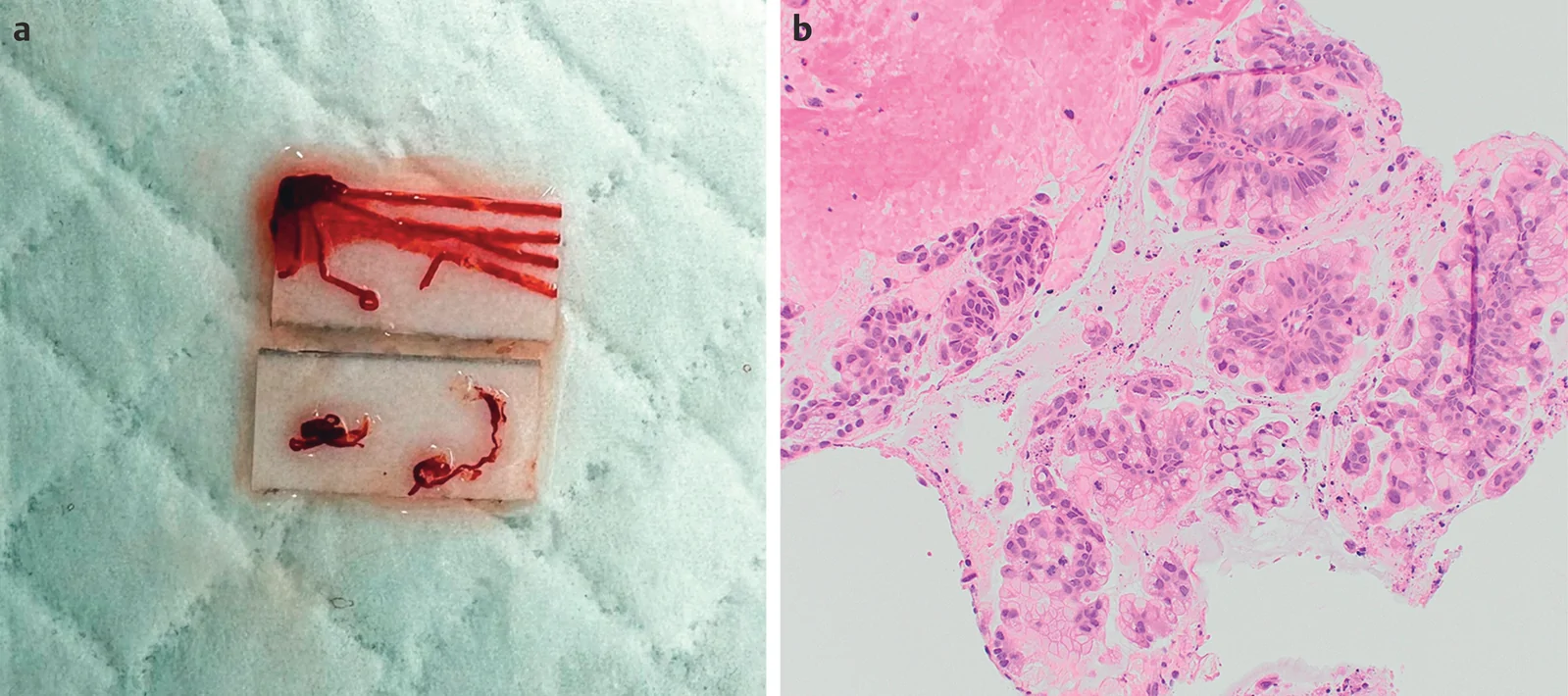
( **a** ) A tissue specimen was obtained by EUS-guided tissue acquisition. ( **b** ) A histopathological image of the obtained specimen (hematoxylin and eosin staining). Papillary proliferation of tumor cells was observed, raising suspicion for pancreatic carcinoma.

The EBUS scope has a distal end outer diameter of 6.9 mm, enabling transoral passage into the stomach. Ultrasound imaging revealed a papillary-growing tumor within the main pancreatic duct in the pancreatic body, with suspected invasion into the surrounding parenchyma ( Fig. 4 ). Using a 22-gauge needle (Visishot2, Olympus, Tokyo, Japan), the hypoechoic lesion, measuring approximately 8 mm in diameter, was punctured twice for tissue acquisition ( Fig. 5a ), and histopathological examination suggested adenocarcinoma ( Fig. 5b ). Based on these findings, neoadjuvant chemotherapy was administered, and distal pancreatectomy was performed 2 months later. The resected specimen confirmed invasive ductal carcinoma with intraductal spread, consistent with the EUS findings.

Only a few case reports have described pancreatic biopsy using an EBUS scope, all performed by pulmonologists in the context of lung cancer staging or evaluation, 2​ ​3​ and its use in the gastrointestinal field remains extremely limited. The EBUS scope has a working length of 60 cm, limiting its use to lesions accessible via the transgastric approach. It also lacks an elevator mechanism, requiring careful needle manipulation. Nevertheless, in cases, where the insertion of a conventional echoendoscope is difficult, an EBUS scope may represent a viable alternative for EUS-TA.

Endoscopy_UCTN_Code_TTT_1AS_2AD
